# Early‐life exposures to infectious agents and later cancer development

**DOI:** 10.1002/cam4.538

**Published:** 2015-09-17

**Authors:** Vidya Vedham, Mukesh Verma, Somdat Mahabir

**Affiliations:** ^1^ Methods and Technologies BranchNational Cancer Institute, National Institutes of Health (NIH)9609 Medical Center DriveRockvilleMaryland20850; ^2^ Environmental Epidemiology BranchEpidemiology and Genomics Research ProgramDivision of Cancer Control and Population SciencesNational Cancer Institute, National Institutes of Health (NIH)9609 Medical Center DriveRockvilleMaryland20850

**Keywords:** Cancer, early life exposure, infectious agents, perinatal transmission

## Abstract

There is a growing understanding that several infectious agents are acquired in early life and this is the reason why available vaccines target the new born, infants, and adolescents. Infectious agents are associated with cancer development and it is estimated that about 20% of the world's cancer burden is attributed to infectious agents. There is a growing evidence that certain infectious agents acquired in early life can give rise to cancer development, but estimates of the cancer burden from this early‐life acquisition is unknown. In this article, we have selected five cancers (cervical, liver, Burkitt's lymphoma‐leukemia, nasopharyngeal carcinoma, and adult T‐cell leukemia‐lymphoma) and examine their links to infectious agents (HPV, HBV, HCV, EBV, and HTLV‐1) acquired in early life. For these agents, the acquisition in early life is from mother‐to‐child transmission, perinatal contact (with genital tract secretions, amniotic fluids, blood, and breast milk), saliva, sexual intercourse, and blood transfusion. We also discuss prevention strategies, address future directions, and propose mechanisms of action after a long latency period from the time of acquisition of the infectious agent in early life to cancer development.

## Introduction

Infectious agents have a natural history and therefore, a life‐course approach to infections and cancer prevention should be an important research priority. While the links between infections and cancer has been known for a long time and estimates suggest that about 18% of the global cancer incidence is attributable to infectious agents 1, understanding of the timing of acquisition of the infectious agent in early life and cancer development later in life is unclear. The main infectious agents are Helicobacter pylori (HP), 5%, human papilloma viruses (HPV), 5%, hepatitis B (HBV) and C (HCV) viruses, 5%, Epstein–Barr virus (EBV), 1%, and human immunodeficiency virus (HIV) plus human herpes virus (HHV) 1%, respectively, of the global cancer burden 1. Other infectious agents that cause cancer include human T‐cell lymphotropic virus type‐1 (HTLV‐1), schistosoma hematobium, and the liver flukes 1. Several infectious agents that are associated with cancer are acquired in early life, during in utero, infancy, early childhood, and adolescence, have a long latency period in human carcinogenesis, but this relationship is largely unexplored.

In this review, we select five cancers (cervical, liver, Burkitt's lymphoma‐leukemia [BL], nasopharyngeal carcinoma [NPC], and adult T‐cell leukemia‐lymphoma [ATL]) to describe the epidemiological links with specific infectious agents acquired in early life, cancer prevention strategies, discuss challenges and opportunities in this field of research, and propose potential underlying biological mechanisms of action. For this article, the period of early‐life exposure to infectious agents is defined as from in utero to childhood/adolescence (0–14 years) and cancer development can be anywhere in the life course. We focus on cervical cancer, liver cancer, BL, NPC, and ATL because from the current literature, these cancers are better understood in terms of early‐life exposures. Today, across the cancer spectrum, a vast majority of cancer prevention effort has been focused on the later part of the median split of the life span, where it is probably less useful or might not be beneficial at all. If indeed, it is established that that early‐life exposures play an important role in later cancer development, whether in childhood or adulthood, this knowledge can be translated to interventions targeting early‐life exposures to infections, where the gains can potentially be more substantial.

## Cervical Cancer

### Epidemiology

Worldwide, in 2012 there was an estimated more than half a million new cervical cancer cases and 266,000 deaths from the disease 2. In the developing world, cervical cancer is the most commonly diagnosed cancer and the third leading cause of cancer deaths among females. Incidence and mortality rates are highest in areas of Africa, Caribbean, and south‐central Asia 2.

HPV infections cause almost all of the cervical cancer cases worldwide 3. HPV is a heterogeneous family of DNA viruses making up more than 100 types of which about 13 are cancer‐causing viruses. HPV infections can also cause skin and genital warts, and respiratory papillomatosis.

HPV infections account for about 5% of the global cancer cases, including cervical, other genital tract, anus, and oropharyngeal 4.

### Early‐life infection

Substantial exposure to HPV occurs during the early life period. HPV is a common sexually transmitted virus and it can be spread during vaginal or anal sex. However, HPV can also be acquired by nonsexual modes of transmission. The virus can also be transmitted from an HPV‐positive mother to her new born 5. In 1978, HPV 1, HPV 2, and HPV 3 were shown to predominate in skin warts in children between 5 and 15 years and HPV 4 in older children 6. The 1978 publication was the first epidemiological investigation of HPV in children. In 1986, the first report of the presence of HPV DNA in the foreskins of asymptomatic neonates was published. In 1989, the first report emerged showing that 48% of newborn children had the presence of HPV DNA who were delivered vaginally from young mothers (mean age = 22.4 years) from a low socioeconomic (SES) group who had detectable levels of HPV DNA in exfoliated cervical cells 7. Perinatal HPV transmission to newborns can occur through exposures to genital tract secretions, amniotic fluids, or blood during vaginal deliveries in mothers with cervical HPV infections 8, 9, 10, 11. Subsequent studies have reported HPV DNA detection rates in mother‐baby pairs ranging from 4% to 87% 10. The 1989 published study 7 and other studies 10, 12 point to an in utero mode of HPV transmission as well.

Several studies have also reported oncogenic strains of HPV in breast milk in a minority of lactating mothers 13. While there is a concern that high‐risk HPV infections might be transmitted during breast‐feeding, this is a very rare event and HPV‐positive mothers are not as a matter of policy advised against breast‐feeding their children. There are also implications for human donor milk banks, but pasteurization methods are available that can completely inactivate high‐risk HPV in human milk 13.

During the adolescent and young adult years, the virus can be acquired from sexual contact and give rise to cervical cancer years later in life after a long latency period. This early life period is very critical in the life course because sexual activity is the primary mode of transmission of high‐risk HPV infection. A high percentage of infected women usually clear their infection by immunological mechanisms 14.

### Prevention

The two approved vaccines against HPV, effective against HPV 16 and 18, the two types known to cause 70–80% of cervical cancers and proportions of anogenital and oropharyngeal cancers, target young populations, and is aimed at preventing acquisition of the virus in early life. A recent systematic review and meta‐analysis of population‐ and clinic‐based studies of the effects of HPV vaccination programs since the start in 2007 showed that the greatest impact is achieved by targeting young age groups with high vaccination coverage. The 19 included studies in the meta‐analysis, all from high‐income countries with high vaccination coverage, showed that HPV 16 and HPV 18 infection and anogenital warts decreased by more than 60% in girls younger than 20 years of age 15. These results imply that the greatest effects could occur from school‐based HPV vaccination initiatives. This could be more challenging in the developing world, where 80% of the global cervical cancers occur, and where lack of resource hinders screening and vaccination efforts.

## Liver Cancer

### Epidemiology

In all parts of the world, primary liver cancer is more common in men than in women. An estimated 782,500 new liver cancer cases and 745,500 deaths occurred worldwide during 2012 2. The estimated new number of cases worldwide was 520,000 and 230,000, respectively, for men and women in 2008 16. Worldwide and in the less developed countries, liver cancer is the leading cause of cancer deaths 2. China alone accounts for about 50% of the total number of cases and deaths 2. About 70–90% of the primary cancer cases worldwide are hepatocellular carcinoma (HCC) 2. HCC is the fifth most common cancer in men and the seventh in women in 2008 16. The rates of HCC is about 2–3 times higher in men than in women and are more pronounced in high‐incidence than low‐incidence areas 16.

About 80 percent of HCC cases are linked to chronic infections with either HBV or hepatitis C virus (HCV), both of which have been categorized by the International Agency for Research on Cancer (IARC) as human carcinogens. HBV is estimated to cause 50–55% and HCV 25–30% of all HCC 17. In addition to its links to HCC, HBV is also a factor in liver cirrhosis. It is estimated that about 2 billion people worldwide have been infected with HBV, while 240–400 million have chronic HBV infection 18. While not an infectious agent, aflatoxin, a mycotoxin produced by fungi of the *Aspergillius* species is also a major risk factor for the development of HCC. Aflatoxin grows on foods such as corn and peanuts when it is improperly stored. Dietary aflatoxin exposure is a major problem in low‐income countries and the highest levels are found in sub‐Saharan Africa and southern China 16. IARC has also classified naturally occurring aflatoxin as a carcinogen.

### Early‐life infection

HBV and HCV are transmitted through exposure to bodily fluids (i.e., semen and blood), and infected individuals have a higher risk of developing HCC 19. Infections from HBV and HCV are also acquired in the early life course. Worldwide, there is a strong geographic correlation between HCC and the prevalence of HBV surface antigen or antibody to HCV 17.

Global variation in liver cancer incidence rates across countries show a shift towards young age groups. The age of onset of HBV and HCV are very important, not only in the natural history, but also in the epidemiology of HCC. In areas where the virus is endemic, the majority of HBV infections are vertically acquired at birth or before the age of 5 20. In high‐risk populations of the world, higher risk of HCC is observed in the younger age groups where HBV infections are acquired perinatally or in early childhood and adolescence. The risk of HCC related to HBV is higher when chronic HBV infection is acquired early in life (0–5 years) than at older age groups 21. In high‐risk regions, such as some Asian countries, there is a high prevalence of HBV surface antigen carriers and remain HBV‐DNA positive throughout their reproductive years, where as in other high‐risk areas such as in parts of Africa, mother‐child transmission is probably lower than child‐to‐child transmission 17.

In geographic regions of low‐risk liver cancer such as in developed countries, acquisition of the HBV infection at birth is rare. In low‐risk regions of the world, liver cancer incidence rates below the age of 40 years is relatively rare 17. In these low‐risk regions, most infections are acquired in adolescence or adulthood through sexual intercourse, blood transfusion, or invasive nonsterile procedures 16. Among immigrant populations, the observed risk reduction seen among immigrants to low‐risk countries, has been described to reflect a reduction in the mother‐to‐child transmission of HBV because of mixing populations, and a reduction in exposure to contaminated blood in medical procedures, decreased prevalence of HBV, and possibly HCV in siblings, and recently the impact of the HBV vaccines 17. Therefore, the high‐risk regions of the world in contrast to low‐risk regions, mother‐to‐child transmission of HBV and HCV is the typical route of acquisition of the virus in early life. In addition to mother‐child transmission of the viruses, some horizontal transmission from infected household contacts also occur 22. According to a recent Cochrane report, it was estimated that the risk for chronic HBV infection to a newborn infant ranges from 70% to 90% by 6 months postpartum when the mother is a HBV carrier 22.

Most HBV‐infected individuals who develop HCC have liver cirrhosis, secondary to chronic inflammation, although HBV can cause HCC in the absence of cirrhosis. In HBV‐positive adults, factors reported to increase HCC risk include excessive alcohol consumption and tobacco smoking, coinfection with HCV or hepatitis D virus, cirrhosis, and environmental exposures to aflatoxin 23. Epidemiological evidence indicates that HCC risk is increased when humans are exposed to both HBV and dietary aflatoxin, and reducing aflatoxin below detectable limits reduce HCC incidence between 14–19% in the general population 16. Coinfection with HBV, HCV, and associated liver disease also is found in ~30% of HIV patients 24. The prevalence of occult HBV infection due to early‐life exposures in HIV patients varies substantially across geographic regions 25, 26. Occult HBV infection in HIV‐positive individuals may induce hepatic flares and lead to cirrhosis and cancer 27, 28, 29, 30.

### Prevention

Since HBV infections are a major cause of HCC, vaccines are the most effective way to prevent HBV infection in most populations 31. Children in the United States get their first dose of vaccine at birth and complete the vaccine series by 6–18 months of age, confirming the importance of targeting early‐life infections. Countries such as the United States and China have developed universal HBV vaccination strategies 32. At the beginning of the 1980s, a plasma‐based vaccine was introduced, followed by a recombinant DNA‐based vaccine. Both vaccines were successful, and vaccination was recommended to all newborns and infants (not just high‐risk individuals) 33, 34. The World Health Assembly has recommended routine infant HBV vaccination in all countries since 1992. So far 177 countries have followed those recommendations 32. In Taiwan, universal HBV vaccination has almost eradicated HBV‐related pediatric liver cancer 35. The average annual incidence declined from 0.70 per 100,000 children (6–14 years of age) between 1981 and 1986, to 0.57 between 1986 and 1990, to 0.36 between 1990 and 1994 36. Correspondingly, the mortality rates of HCC also declined, showing the success of implementing this vaccination program. A 20‐year follow‐up showed that HCC incidence rates were significantly lower in children (6–14 years) who were vaccinated compared to those unvaccinated at birth 37. In addition, it has been well established that HBV vaccination during pregnancy is safe and provides passive transfer of maternal antibodies to the newborn 38, 39, 40, 41, 42. Currently, there is no vaccination for HCV. Interferon treatment is effective in clearing acute HCV infections in about a third of the patients 43. The universal hepatitis B vaccination in Taiwan appears to have decreased the incidence of HCC in children from 1981 to 1994 44.

Strategies to prevent HCC vary depending on the mode of transmission and the infectious agent. In developed countries, HCC is predominantly found in intravenous drugs and HIV‐positive individuals 45. Therefore, a public health effort to educate these individuals can help prevent them from getting infected 46. In addition, surgical resection or liver transplantation are also options against HCC 47.

In the subtropical regions of the world such as sub‐Saharan Africa, aflatoxin, a naturally occurring mycotoxin produced by many species of fungus, infects foods such as peanut and maize. When aflatoxin‐infected foods are consumed, they are metabolized by the liver to a reactive epoxide intermediate implicated in the development of HCC, especially in individuals with either HBV or HCV infection 48. Usually, early‐life exposures to aflatoxins are more serious as a driver of cancer. More than 4 billion people worldwide are exposed to dietary aflatoxins 49. It is estimated that aflatoxin exposure may account for 5–28% of total HCC cases worldwide 48, 50. Hence, while it is not an infectious agent, prevention of aflatoxin‐induced HCC is an important strategy for preventing HCC. In economically developing countries, a number of strategies may be applied. These include the development and augmentation of cancer surveillance, food monitoring, and public health response capacity of the affected regions; quantification of the health impacts and burden of cancer due to HBV, HCV, and aflatoxin exposure; and evaluation of the efficacy of intervention strategies. This would require the commitment of sufficient resources and the collaboration in a country's public health sectors at the local, regional, and national levels as well as the agricultural sectors for linkages to aflatoxin.

## Burkitt Lymphoma‐Leukemia

### Epidemiology

BL is the most common form of non‐Hodgkin lymphoma (HL) in children and adolescents 51. Two important epidemiological correlates to the pathogenesis of BL are the geographical association with malaria and early infection by EBV 51. Dr. Denis Burkitt, working in Africa was the first to discover BL. There are three broad categories of BL: (1) Endemic BL, (2) sporadic BL, and (3) HIV‐associated BL 52. Endemic BL is also called the African type because of its high incidence in certain parts of Africa. BL in other parts of the world are referred to as “sporadic.” In 1982, it was observed that patients with HIV were also predisposed to non‐HL, including BL 51. The different subtypes of BL and their associated causative infectious agents are described below.

EBV, also called HHV‐4 is a member of the herpes virus family and one of the most common human viruses that infects about 90% of adults worldwide 53, 54. Endemic BL is the most common childhood malignancy etiologically associated with EBV (EBV) infection, and accounts for up to 75% of all childhood malignancies 55. Endemic BL has a high incidence in equatorial Africa and has a 95% rate of association with EBV infection. Endemic BL is often linked to coinfection with the malaria‐causing bacterium *Plasmodium falciparum* 56. Sporadic BL occurs worldwide but is less frequently associated with EBV infections 57. In the United States, only 15–30% of the cases of sporadic BL are associated with EBV infections 52. Incidence of sporadic BL is significantly increased when associated with HIV infections 58. BL is a highly aggressive and often life threatening disease, however, it is often considered a curable form of lymphoma 59, 60. Interestingly, about 30–50% of HIV‐associated cases of BL are EBV positive 61. EBV‐associated lymphoma is often thought to be caused by a defective immune response against EBV in HIV‐positive individuals 61. HL accounts for ~9% of all childhood cancers 62 and is a potentially curable lymphoma characterized by the expansion of cells of the B‐lineage called Reed–Sternberg cells 63. EBV infections are associated with about 30–50% of HL and the EBV incidence rate for HL increases to about 95% in AIDS patients 64. The incidence of EBV‐positive Hodgkin disease is age‐related and preferentially associated with pediatric and older patients 65. In Central and South America, up to 70% of pediatric HL is associated with EBV 66, 67.

### Early‐life infection

EBV was first isolated from tissue samples of BL in African children 54, 68 suggesting that early‐life exposure is an important timing of the infection. In most of the less developed countries, the initial EBV infection usually occurs in the first decade of life as an asymptomatic infection, establishing its presence in young children. In the more developed countries, the initial EBV infection occurs primarily in adolescence or in adulthood, accompanied by infectious mononucleosis (IM) in about 50% of the cases 69.

The primary mode of EBV infection is through saliva and/or oral contact and genital secretions 70. Children infected with EBV are asymptomatic or have mild symptoms that are indistinguishable from those of other childhood illnesses (severe diarrhea, vomiting, ear infection, persistent cough, mouth infection, eye infection, influenza, and unspecified “other infections”). In infants, EBV infection often occurs as soon as maternal antibody protection (present at birth) disappears. The prevalence of EBV varies widely; young adults in lower socioeconomic populations exhibit higher rates of primary EBV infection. Regarding geographic and age distribution of EBV, the age at primary infection varies substantially. While more than 80% of children in Uganda are estimated to be seropositive for EBV by age one, this estimate is only ~45% in the rural United States of America. Furthermore, EBV infection during early childhood is usually subclinical, a delay in acquiring a primary EBV infection at an older age in childhood or adolescence (more common in developed countries) can manifest as IM occurring in ~25–75% of EBV‐infected people. The distribution of acute EBV infection by age showed peaks at the age 2–4 and 14–18 years 71. Host susceptibility to EBV‐related tumors can differ depending on geographical and immunological variation in the populations. EBV often establishes a lifelong dormant infection in most infected individuals and the virus is shed intermittently in the saliva of healthy carriers. Since the shedding of virus occurs randomly, it is almost impossible to prevent person‐to‐person transmission of EBV.

Furthermore, EBV infection during early childhood is usually subclinical, a delay in acquiring a primary EBV infection at an older age in childhood or adolescence (more common in developed countries) can manifest as IM occurring in ~25–75% of EBV‐infected people. The distribution of acute EBV infection by age showed peaks at the age 2–4 and 14–18 years 71. Host susceptibility to EBV‐related tumors can differ depending on geographical and immunological variation in the populations. EBV often establishes a lifelong dormant infection in most infected individuals and the virus is shed intermittently in the saliva of healthy carriers. Since the shedding of virus occurs randomly, it is almost impossible to prevent person‐to‐person transmission of EBV. EBV infections in early life are associated with many cancers later in life such as Burkitt lymphoma (BL), HL, NPC, and post‐transplantation lymphoproliferative disease 72.

For HIV‐related infections, worldwide over 260,000 children were infected with HIV in 2012. It is estimated that the majority of these infections were from mother‐to‐child transmission, which can take place when the child is in utero, during labor and delivery, or from breast‐feeding 73. This mother‐to‐child transmission continues to add to the global burden of HIV‐related illnesses despite advances in antiretroviral medications.

### Prevention

Development of a prophylactic vaccine is the most important future step toward controlling primary EBV infection because some aspects of EBV infection are under immune control and vaccine‐induced immune response may be able to regulate primary infection and/or modify subsequent persistent infection 74. Morgan et al. emphasized that most primary infections occur in first few years of life and prophylactic vaccine is the appropriate prevention strategy 74. Most of the EBV vaccine development efforts have focused on a glycoprotein abundant on the viral envelope to generate neutralizing antibodies 75. The target of these vaccines should be young children so that vaccine‐induced mucosal or systemic immune response may prevent primary infection or existing infection 76. In February 2011, the National Institutes of Health provided guidelines for future EBV vaccine development that focus on preventing EBV‐associated disease (rather than treating infection) by identifying disease‐predictive surrogate markers 77.

The high prevalence of EBV and its easy mode of transmission make it an extremely difficult target for primary prevention. Furthermore, the limitations in understanding the oncogenetic process and in identifying the factors that trigger cancer initiation after EBV infection complicate the development of targeted prevention strategies 78. Currently, broad‐spectrum antiviral agents such as ganciclovir or acyclovir (immune‐based therapies) have shown some success when given at early stages of life in suppressing virus replication and virus shedding during diseases characterized by acute or lytic replication of EBV 76.

## Nasopharyngeal Carcinoma

### Epidemiology

NPC is a unique squamous cell carcinoma of the head and neck 79. NPC is currently classified as keratinizing carcinoma or squamous cell carcinoma, nonkeratinizing carcinoma, including differentiated and undifferentiated types, and basaloid squamous cell carcinoma 80. NPC represents virtually all nasopharyngeal cancers. Worldwide, NPC is a rare cancer and in 2008 there were ~84,400 incident cases and 51,600 deaths 81. However, there is substantial geographic variation in NPC incidence rates. Rates are high in the Arctic among Inuits and Aleuts, North Africa, and in southern Asia 80. NPC is endemic in southern China and accounts for about 20% of adult cancers in this region 82. Undifferentiated NPC mostly affects individuals in their mid‐40s and is highly prevalent in Hong Kong, Taiwan, and the Inuits in Alaska and Greenland 83. Several nonviral environmental factors such as smoking, heavy alcohol consumption, high intake of salt‐preserved and fermented foods have been associated with NPC development 84.

The high incidence of NPC in China and southern Asia is attributed to the nonkeratinizing NPC subtype that is predominantly associated with EBV 80. DNA samples from patients with undifferentiated NPC in high, intermediate, and low incidence areas were consistently positive for EBV 79. There is emerging evidence that HPV may also be important in NPC development 80. For example, several studies have isolated HPV from NPC tumor tissues with weighed prevalence of up to 31%.

### Early‐life infection

In areas where NPC is endemic, it is possible that EBV infection in early life plays an important role 85. EBV infection in early life has a long latency period till NPC onset and it is believed that other factors may contribute to the ultimate development of NPC 85. Acquisition of the EBV in early life is expected to affect NPC development in a similar manner to the other EBV‐related cancers described in this article. There is also emerging evidence linking HPV to NPC, but it remains to be established. HPV is established to be the major causative agent for cervical cancer and the cellular similarity of the cervix with the nasopharynx (both covered by squamous epithelium) may imply that HPV could have similar infection behavior in the nasopharynx. HPV is linked to head and neck cancers, for example, a recent meta‐analysis reported that HPV DNA positive is found in a high proportion of oropharyngeal squamous cell carcinomas (45.8%) 86. The meta‐analysis also showed that HPV16 was the most important oncogenic HPV‐type accounting for more than 80% of all HPV‐positive head and neck squamous cell carcinomas 86. However, the observed links between HPV to NPC presents intriguing questions regarding the original mode of transmission of HPV in NPC. Chan et al. 87 have proposed that perinatal nasopharyngeal infection of HPV as the important etiological factor in NPC development. This idea is based on emerging evidence that perinatal HPV transmission to newborns can occur through exposures to genital tract secretions, amniotic fluids, or blood during vaginal deliveries in mothers with cervical HPV infections 8, 9, 10, 11. Because HPV exposure is a strong risk factor for head and neck cancers, it is quite possible that increases in oral sex in early life periods such as in adolescence or young adulthood, may contribute to a part of the burden of NPC. However, the evidence to support this proposition is not yet available.

### Prevention

In addition to EBV and HPV, the most important risk factor for NPC is the consumption of salt‐preserved fish, which is a staple diet in several areas where NPC is endemic 85. Other types of salt‐preserved foods, and lack of adequate consumption of fruits and vegetables also increase risk for NPC. Experimental studies in rats have confirmed the carcinogenicity of salt‐preserved fish. These experiments have shown that rats consuming high quantities of salt‐preserved fish develop NPC 85. Apparently, the salt preservation technique is not done very well, allowing some sort of reaction in which carcinogenic nitrosamines and bacterial mutagens develop 85. The most useful way to prevent some of the NPC would be from the reduction in consumption of salt‐preserved fish.

## Adult T‐Cell Leukemia‐Lymphoma

### Epidemiology

ATL is a malignancy of the T‐lymphocytes caused by HTLV‐1. HTLV‐1 is now estimated to infect ~15–20 million people worldwide 88. Similar to EBV‐infected individuals, about 90% of HTLV‐1‐infected individuals are asymptomatic. HTLV‐1 is endemic to southwestern Japan, Africa, the Caribbean islands, and South America is becoming areas of high prevalence 89, 90. In Japan, ~1 million people are carriers of HTLV‐1. The incidence of ATL among HTLV‐1 carriers in Japan is about 60 per 100,000 individuals 91. HTLV‐1 is very rare in North America. Seroprevalence is low in North America and Europe where HTLV‐1 infection is mainly found in immigrants from endemic areas 90. In Africa, the HTLV‐1 seroprevalence rate is rather high with about 2% of the population infected, especially in sub‐Saharan countries 91. In Central and South America, Brazil has the highest HTLV‐1 seroprevalence rate, with about 1% of the population infected 91.

### Early‐life infection

HTLV‐1 is firmly established as the causative virus for ATL, but studies have shown that HTLV‐1 alone is insufficient to develop ATL. It is also now clear that ATL develops in adults after a long incubation period of about 20–30 years 91. The most probable cause of ATL may be HTLV‐1 infection early in life up to childhood 92, 93, 94. HTLV‐1 infection occurs from contact with bodily fluids containing infected cells 95. There is evidence that the major routes of transmission of the virus is from mother to child, sexual intercourse, and blood transfusion containing HTLV‐1‐infected lymphocytes 96. The most common mode of HTLV‐1 transmission in children is the transfer of infected maternal lymphocytes from infected mothers through breast milk 97. HTLV‐1 can be transmitted via breast milk to the child and significantly increased HTLV‐1 infection rate is seen in breast‐fed children compared to bottle‐fed children. Long‐term prospective data from a Japanese study show that mother to child transmission rates were 20.5% in infants breast‐fed for 6 months or more, 8.3% in those breast‐fed for <6 months and 2.4% in formula‐fed infants exclusively 98. Family history of ATL is an important risk factor for ATL. There is also evidence that ATL occurs when HTLV‐1 infection occurs in childhood, but it rarely occurs when HTLV‐1 infection is in adulthood 91. Therefore, early life acquisition of HTLV‐1 is an important feature in adult development of ATL.

**Table 1:** Summary of the five cancer types, associated infectious agents, types of early‐life transmission, and possible prevention strategies

Cancer	Infectious agent	Types of early‐life transmission	Prevention strategy (targeting infections only)
Cervical	HPV	Sexual intercourse—adolescent and young adultPerinatal—genital tract secretions; amniotic fluids; blood during vaginal deliveriesBreast milk	HPV vaccination in childhoodCervical cancer screeningUse latex condoms when having sexSex education programs targeting adolescents and young adults
Liver	HBV, HCV	HBV and HCV:Mother‐to‐child transmission at birthSexual intercourse (adolescent and young adult)Blood transfusionExposure to blood from infected individuals	HBV vaccination to newborns and infantsScreen bloodSex education programs targeting adolescents and young adults
Burkitt lymphoma‐leukemia	EBVHIV	Saliva—oral contactGenital secretionsBloodMaternal‐to‐child	Vaccine development against EBVAvoid kissing or bodily contact with secretions from known infected individuals
Nasopharyngeal	EBVHPV	Same for EBV and HPV	Same for EBV and HPV
Adult T‐cell leukemia‐lymphoma	HTLV‐1	Mother‐to‐childBreast milkSexual intercourseBlood transfusion	Use latex condoms when having sexAvoid bodily contact with secretions from known infected individualsIn endemic areas, screen pregnant women to recommend whether to breast‐feed or bottle‐feedScreen blood supply

HPV, human papilloma virus; HBV, hepatitis B virus; HCV, hepatitis C virus; EBV, Epstein–Barr virus; HIV, human immunodeficiency virus; HTLV‐1, human T‐cell lymphotropic virus type‐1.

### Prevention

Primary prevention seems to be a feasible strategy to reduce ATL disease burden. Screening of donated blood for transfusion for the presence of HTLV‐1, which started since 1986 in Japan, has almost eliminated transmission via transfusion 96. This method should be implemented in other endemic areas of the world as well. Sexual transmission is primarily from male to female through HTLV‐1‐infected lymphocytes in the semen 96. Sexual transmission can be prevented by the use of latex condom. In Japan, starting 2011, to prevent mother to child transmission, all pregnant women are screened for HTLV‐1 infection and recommendations are made for either exclusive formula feeding or breast‐feeding for a maximum of 3 months if the mother is infected 96. Similar policies should also be implemented in other endemic areas of the world. Educating HTLV‐1‐infected mothers about possible risks was effective in reducing transmission in regions of Japan where the virus is endemic 99. A major susceptibility locus of HTLV‐1 infection in childhood maps to chromosome 6q27; a specific allele at this locus confers a predisposition to childhood HTLV‐1 infection 100. Genomic studies may help identify the molecular mechanisms of HTLV‐1 infection, and thereby lead to the development of targeted prevention strategies.

Since emerging evidence indicate that HTLV‐1 infections are most likely acquired early in life, efforts to develop vaccines have targeted young individuals. Asymptomatic carriers of HTLV‐1 are known to have neutralizing antibodies 101 that protect against the virus in children. Maternally acquired antibodies protect infants from HTLV‐1 infection in the early months of life 88. Currently, candidate vaccines against HTLV‐1 are being tested in immunized squirrel monkeys 102. Broad‐spectrum antiviral agents such as ganciclovir or acyclovir (immune‐based therapies) have shown some success when given at early stages of life in suppressing virus replication and virus shedding during diseases characterized by acute or lytic replication of EBV 76.

## Future Directions: Challenges and Opportunities

While emerging evidence shows that infections in early life increase risk for cancer development later in life, few well‐defined epidemiological studies on this topic have been conducted, largely because of the methodological challenges of conducting prospective cohort studies, the long latency period of the infections, the years of follow‐up required, the difficulty of biological specimen collection, and the high costs associated with prospective studies 103, 104, 105, 106. In this review, we present evidence showing that early life exposures to specific infectious agents (HPV, HBV, HCV, EBV, HIV, and HTLV‐1) are associated with either cancer development in childhood or adulthood (Table 1). However, even more complicated is the knowledge that while specific infections in early life are associated with the development of specific cancers, there is also emerging evidence that generalized early life infections may be protective against the development of other cancers 107. Recently, Urayama et al. examined the relationship between surrogate measures of early life exposures to generalized infections assessed by daycare attendance, birth order, and common childhood infections in infancy with the risk of acute lymphoblastic leukemia (ALL) and found evidence that generalized infections may be protective 108. This study concluded that when the three measures were evaluated separately, daycare attendance by the age of 6 months and birth order were associated with a reduced risk of ALL among nonHispanic white children, but not Hispanic children. Interestingly, there was evidence of a protective role for infection‐related exposures early in life in both the nonHispanic white and Hispanic populations 108. The biological basis for the distinction between infectious agents that increase the risk of developing cancer later in life, versus those that decrease this risk, remain unknown and should be investigated. This is important because according to the microbe hygiene hypothesis, if there is insufficient beneficial microbe exposure during perinatal life that should coevolve with humans then immunoregulation would be adversely affected 109. Certain types of infections may also reduce cancer risk by boosting the host's immune system.

It is apparent that in most cases, early‐life acquisition of the infectious agents alone is insufficient for cancer development. While chronic inflammation may play important roles in driving infections acquired in early life to oncogenesis 109, this remains unresolved, as well as other cofactors. Chronic proinflammatory factors can initiate carcinogenesis via increased DNA damage and defective repair, but microbial and nonmicrobial factors such as diet modulate inflammation. Therefore, detailed characterization of perinatal, childhood, adolescent, and young adult acquisition of infectious agents, other microbes, and nonmicrobial modifiable factors would be important in understanding diseases such as Burkitt's lymphoma, liver cancer, adult T‐cell leukemia, cervical cancer, nasopharyngeal cancer, and others. An important hindrance to the characterization of the infections acquired in early life, such as from maternal‐fetal transmission, is the high cost of screening methods. A recent invention, published in 2015, may make it possible to test for current and past infections with any known human virus by a multiplex reaction using a single drop of a person's blood 110. The method, called VirScan, is sensitive and selective, and although it has few limitations, can be an efficient alternative to existing diagnostics that test for specific viruses one at a time. VirScan is reported to be cheap and particularly suited to epidemiological studies 110.

From a public health perspective, the greatest impact would come from policies aimed at improving sanitation to prevent early‐life carcinogenic infections. Infections can be prevented by improved sanitation, water, food, and air quality, as well as clean living quarters 111. Similarly, educating the public about the parenteral and sexual transmission routes may be effective methods of primary prevention in areas where the disease is endemic 8, 10.

An important research priority would be to improve understanding of the mechanisms by which specific early‐life infectious agents affect cancer development. This insight can be helpful for initiatives to target therapeutic and preventive strategies. If indeed, it is established that early‐life exposures to infections play a major role in later cancer development, whether in childhood or adulthood, this knowledge can be translated to interventions targeting early‐life exposures to infections to attenuate the cancer burden. This is illustrated by the success of HPV vaccination in cervical cancer prevention. The use of vaccines against cancer‐associated pathogens is a promising area of primary cancer prevention research. There is a growing demand for cheaper and alternative vaccines, especially in populations of low socioeconomic background where infection rates are higher 112, 113.

We hope that further research will be encouraged to explore the role of infections acquired in early life and cancer risk later in life because it has important public health significance in its potential to both prevent and treat the relevant cancers. An important need is proper epidemiological study designs and methods to study both the acquisition and consequences of early‐life infections in terms of cancer etiology. Large‐scale prospective studies with adequate sample sizes are need. Birth cohorts that are now starting with longitudinal follow‐up would be ideal opportunities to start collecting biological samples for such investigations. Specific funding opportunities to support such initiatives would also be required.

### Mechanistic insights

Theories about the roles of biological factors such as genetics and epigenetics as mediators of early‐life infections and later cancer development have been proposed, but the relationship is complex and requires substantial investigation because of the lack of adequate data 114, 115, 116, 117, 118, 119.

**Figure 1:**
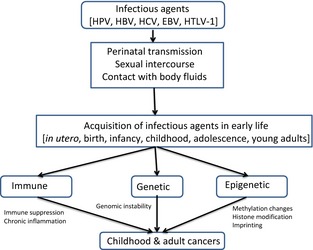
Basic mechanistic model of the links between early‐life exposures to infectious agents and cancer.

Three basic mechanisms may explain how early life exposures to infections affect cancer development: (1) epigenetic, (2) immune, and (3) genetic (see Fig. 1).

Probably, most importantly, infectious agents acquired early in life can cause epigenetic changes in host cells and contribute to cancer initiation. Cancer cells are characterized by multiple epigenetic alterations including DNA methylation and histone modification. Epigenetic footprints from exposures to early life infections, as early as during in utero growth, can follow the individual to adulthood 120. DNA methylation and histone modification can regulate gene expression by chromatin remodeling. This can affect genomic stability, influencing carcinogenesis from initiation through progression, from acquisition in early life throughout a person's lifespan, and in some cases across generations, maintained through replication. In early life because of rapid development there is a higher rate of cellular replication and the phenotypic impact may be amplified. At the embryonic stage, the genome of all cells is the same but epigenomic marks are different. During embryogenesis, epigenetic reprogramming occurs and this is the stage which is very sensitive to environmental changes including exposure to infectious agents 120, 121, 122. Epigenetic marks can occur in the placenta and other transient structures during pregnancy and subsequent development of organs during postnatal growth 120, 123, 124. Cells of the developing placenta undergo extensive de novo methylation and placental function changes during gestation 125, 126, and early life infections could affect this cellular machinery leading to cancer. It is now well‐established that methylated genes are turned off; therefore, a loss of DNA methylation can cause abnormally high gene activation. Similarly, hypermethylation of certain genes can inhibit the protective effect of tumor suppressor genes. Understanding the epigenetic alterations by which early‐life exposures to infections affect cancer development is crucial to efforts aimed at tackling the cancer burden. Several groups have shown that viral oncoproteins induce expression and interact with cellular DNA methyltransferases as well as histone‐modifying enzymes such as histone deacetylases and histone acetyltransferases, thereby altering the host cell gene expression 127, 128, 129. Similarly, studies also have shown a high frequency of hypermethylation of the cell cycle regulating gene p16INK4a in HBV‐, EBV‐, and HPV‐related tumors 130, 131. Once integrated into host DNA, these infectious agents can block the host defense mechanisms responsible for the inactivation of integrated foreign genetic material by DNA methylation and thus establish latent infection 132, 133. In a recent review on virus‐induced epigenetic mechanisms related to cancer, Poreba et al. listed the interactions between oncogenic viral proteins and host epigenetic machinery 134. In the case of EBV, host‐driven methylation of the viral genome, overexpression of DNA methyltransferases by latent proteins, three dimensional conformational change in the genome and histone modifications contribute to cancer development 135. Another team of investigators suggested that latent EBV initiates a chain of events involving epigenetic mediated repression of proapoptotic gene *Bim* leading to B‐cell lymphoma 136.

Immune responses can be either immune suppression due to the infectious agent, induction of inflammatory cytokines, or combination of both, secondary to the infection. It has been well‐established that persistence of viral, bacterial, or parasitic infections with resulting tissue damage from host inflammatory responses may predispose the host towards neoplastic transformation 137. Moss and Blaser hypothesized that chronic inflammation due to prolonged infections result in adaptive responses in host epithelium leading to selective survival of abnormal cell growth and malignant transformation 138. An in‐depth understanding of the immunopathogenesis of early‐life infections can be critical to developing effective methods of cancer prevention.

In cancers associated with malignant infections, genetic mechanisms of oncogenesis often involve virus‐induced transformation of host cells. For example, some studies on HPV infections have shown that the viral genome is physically integrated into host cell chromosomes 139. This integration of HPV DNA is a crucial step in carcinogenesis since it promotes the clonal selection of the HPV transformed cells resulting in the development of invasive cervical cancers 140. Further, aging‐related processes that affect the genome are important factors that contribute to the increased risk of developing cancer from early‐life infections. These aging‐related factors include increased susceptibility to DNA damage due to age‐related decline in DNA repair responses and the cumulative exposure to cofactors 141. Infectious agents have evolved unique mechanisms, such as degradation of repair proteins, to circumvent the host repair pathways 142. Infectious agents also can cause genetic mutations in the host genome, including chromosomal translocations, which lead to altered gene expression and neoplastic transformation 143, 144.

While epigenetic, immune and genetic factors, individually and in combination orchestrate the path from acquisition of the infectious agent in early life down to cancer, it is probably much more complex. As discussed in the previous section, it has been well‐established that the infectious agents may be necessary, but not sufficient, for carcinogenesis and often require additional triggers from various endogenous and exogenous agents/cofactors 32, 54. Hence, studies that elucidate mechanisms should also incorporate these additional “triggers”.

## Conflict of Interest

None declared.
